# Language Processing as Cue Integration: Grounding the Psychology of Language in Perception and Neurophysiology

**DOI:** 10.3389/fpsyg.2016.00120

**Published:** 2016-02-16

**Authors:** Andrea E. Martin

**Affiliations:** Department of Psychology, School of Philosophy, Psychology and Language Sciences, University of EdinburghEdinburgh, UK

**Keywords:** language comprehension, sentence processing, cue-based retrieval, cue integration, neurobiology of language

## Abstract

I argue that cue integration, a psychophysiological mechanism from vision and multisensory perception, offers a computational linking hypothesis between psycholinguistic theory and neurobiological models of language. I propose that this mechanism, which incorporates probabilistic estimates of a cue's reliability, might function in language processing from the perception of a phoneme to the comprehension of a phrase structure. I briefly consider the implications of the cue integration hypothesis for an integrated theory of language that includes acquisition, production, dialogue and bilingualism, while grounding the hypothesis in canonical neural computation.

## Introduction

Despite major advances in the last decades of language research, the linking hypothesis between ever-more plausible neurobiological models of language and ever-better empirically supported psycholinguistic models is weak, if not absent. Moreover, we are struggling to answer, and even to ask well, questions like *why is language behavior the way it is? How is language processed? What is “processing difficulty?” What is the source of difficulty in psychological and neurobiological terms*? *What can it tell us about the computational architecture of the language system?* These questions, however frustratingly difficult, speak to our persistent awe at the fact that we humans flap our articulators, we move the air, and in doing so, stimulate formally-describable complex meaning in the heads of other people. And then those people usually do it to us back. So how do we, or rather, our brains, do it?

There must be a good reason for the weak link between psycho- and neurobiological theories of language—namely that it is really hard to find a concept that would be explanatory on multiple levels of analysis in cognitive science (see Marr, 1982). Questions like *what makes language the way it is* probe the computational level of Marr's tri-level hypothesis, asking what the system's goal is, what computation is being performed and to what end. Questions like *how does the system do it* occur at the algorithmic level, asking what the nature of the mechanism that carries out the computation is. Recent debates in cognitive science have cast these two kinds of questions in opposition, or at least, in opposing theoretical camps. Bayesian modelers of perception and cognition form the statistical *what* camp, and non-Bayesians the mechanistic *how* camp (Jones and Love, 2011; Bowers and Davis, 2012). The *what* camp is purportedly less interested in how the mind “does it,” but is focused on reverse engineering how the natural world (or the statistics that describe it) makes cognition the way it is. The *how* camp purportedly wants to uncover the mechanism that the mind/brain uses, instead of a statistical approximation (Jones and Love, 2011; Bowers and Davis, 2012). I will argue that any model of language computation must answer both *how* and *what* questions, and the best model will most likely include both mechanistic and probabilistic elements. The model articulated here asserts a mechanistic psychological operation over representations derived via Bayesian inference (or an approximation there of), which are represented by neural population codes that are flexibly combined using two simple canonical neural computations: summation and normalization.

Rather than trying derive novel psychological mechanisms specific to language, I will ask whether insights from perception and psychophysiology can inform process models of psycholinguistic theory to try to explain why language behavior is the way it is *and* how formal linguistic representations might be extracted from sensory input and represented by the brain. First, I will briefly consider two recent advances in psycholinguistic theory, the Cue-based Retrieval framework (CBR) and Expectation-based parsing (EBP), which have shaped the field in the last decade. Then I will briefly explore the implications of sensory processing models in order to argue that the main insights of these frameworks can be transferred to psycholinguistics as a single mechanism derived from neurobiological principles. Then I will attempt to apply this principle to sentence comprehension, and briefly explore its implications for production, dialogue, language acquisition, and bilingualism. Finally, I will try to deliver predictions that could falsify this approach.

### Two influential theories: cue-based retrieval and expectation-based parsing

The cue-based retrieval framework offers an account for processing difficulty in language comprehension that is based on architectures and mechanisms from human memory, specifically recognition memory (McElree, 2000; McElree et al., 2003; Lewis et al., 2006). It originates from the classic insight that retrieval from memory might be needed to form grammatical interpretations, especially for syntactic structures where words that form a linguistic dependency are separated from each other by other words (Miller and Chomsky, 1963). Quite naturally then, CBR has focused on non-adjacent dependencies of different kinds, mostly subject-verb dependencies (McElree, 2000; McElree et al., 2003; Lewis et al., 2006; Van Dyke, 2007; Wagers et al., 2009; Van Dyke and McElree, 2011; Tanner et al., 2014) but also pronouns, ellipsis and other situations with referential or anaphoric consequences (Foraker and McElree, 2007; Martin and McElree, 2008, 2009, 2011; Xiang et al., 2009; Martin et al., 2012, 2014; Dillon et al., 2013; Jäger et al., 2015).

The appeal of the cue-based framework is the parsimony of explanation—language behavior is the way it is because of the architecture of human memory. Memory is *content-addressable*^1^, or organized by content, and therefore is highly susceptible to interference (see McElree, 2000; McElree et al., 2003; Lewis and Vasishth, 2005; Lewis et al., 2006; and see McElree, 2006; Van Dyke and Johns, 2012, for reviews). Interference occurs when the link between the cues used at retrieval and the intended target representation is not diagnostic (McElree, 2000, 2006; McElree et al., 2003; Martin et al., 2012). Therefore, according to CBR, processing difficulty in language comprehension is due to interference^2^, or more specifically, *cue overload*, the term for the situation when the cues at retrieval are insufficient to elicit the needed representation (McElree, 2006; Van Dyke and McElree, 2011; Van Dyke and Johns, 2012). Whether cue overload arises purely due to similarity between representations and cues, or whether distinctive items in memory are somehow disruptive during retrieval, is an on-going challenging question (see Jäger et al., 2015, for an overview on effect reversals for pronouns). Another important architectural assumption of CBR is that retrieval speed is constant, so effects on performance (either accuracy or reaction time) arise from differences in representation, namely cue-target match vs. the match of the cue to other items in memory (McElree, 2006; see Nairne, 2002 for more on diagnostic cues). Additionally, representations appear to be retrieved without a serial or parallel search (see Townsend and Ashby, 1983; McElree and Dosher, 1989, 1993; Martin and McElree, 2009, for details on how parallel search is falsified). CBR has been well-implemented: Lewis and Vasishth (2005) and Lewis et al. (2006) describe compelling symbolic models of parsing implemented with only one additional parameter than the standard ACT-R model (Anderson, 1983).

Expectation-based parsing has focused on modeling classic sentence processing phenomena (syntactic ambiguity resolution and relative clause processing asymmetries) in a Bayesian framework (Hale, 2001; Levy, 2008, 2013; Smith and Levy, 2013). The approach aim to predict which parts of a sentence will be more difficult to process as reflected in behavioral measures. It marks a renaissance for the role of expectation and its formalization in psycholinguistic theory (cf. MacDonald et al., 1994; Altmann and Kamide, 1999; DeLong et al., 2005; Van Berkum et al., 2005). In EBP, parsing decisions are based on probabilities built up from prior experience, and difficulty stems from the violation of word-by-word expectations of syntactic structure. In other words, the main claim is that surprisal, or the degree to which expectations are not met, is the best predictor of reading time slow down and therefore, of processing difficulty (Hale, 2001; Levy, 2008). This striking insight has a lot in common with ideal observer models of perception, which I will review shortly, by virtue of the fact that both are rational and formalized with Bayes' rule. EBP continues the tradition of frequentist accounts of parsing (e.g., MacDonald et al., 1994) and statistical learning in psycholinguistics (e.g., Charniak, 1996; Saffran et al., 1996; Tabor et al., 1997; MacDonald and Christiansen, 2002). EBP's advantage over previous statistical learning accounts might be that it is formalized with a probabilistic grammar and can be highly predictive of which parse or *where* in a structure difficulty will be encountered (Levy, 2008).

### Challenges for CBR and EBP

Each of these approaches is motivated by the central challenge of parsing: incorporating incoming, new information (phonemes, syllables, morphemes, or lexical items) into a continuously unfolding complex representation. Each approach brings an important insight from a related areas of cognitive science to bear on language processing: (1) for CBR, the parsimony of ACT-R principles and the explanatory concepts of cues and interference, and (2) for EBP, the vital importance of prior experience and expectations, and of formalizing uncertainty. Despite these important insights, the architectural claims that each approach makes are not fully articulated. CBR and EBP might tell us about how an aspect of language processing is carried out, but many questions remain about the nature of the representations and mechanistic processes that are at stake.

The beauty of CBR is that its principles are independently motivated by the architecture of human memory. But despite this, many issues still need to be resolved. First, the psychological mechanism that the additional CBR parameter might correspond to would need to be hypothesized about and tested. Larger architectural questions persist, such as whether retrieval is identical during lexical access and dependency resolution, and whether additional mechanisms besides retrieval might be needed for a fully specified model of parsing. More fundamentally, if grounding language processing in memory processes is what gives explanatory power, then difficult issues about memory processes, such as whether encoding and retrieval ever really separate, need also be addressed. Similarly, complex questions about cues remain: why some representations function as cues and other not, how cues are learned and represented, and how their weights are determined, and whether those weights are determined dynamically all need to be established. The *how* questions might be clearer in CBR, but the answer to *what* questions is offloaded onto memory research.

Similarly, though Levy and colleagues have exacting predictions as to where in a sentence reading slow down will occur, EBP's explanation for “processing difficulty” is not psychological or mechanistic in nature. It is computationally descriptive: re-ranking of probability distributions regarding expected input. Re-ranking of probability distributions actually has a neurophysiological appeal, but is not yet a psychological concept. Since EBP focuses on capturing extant behavioral data patterns and predicting patterns of reading slow down, rather than deriving representational states and processing mechanisms that are both neurobiologically and psychologically plausible, it is not clear how EBP would answer *how* questions. Simply put, EBP is not a process model. Architectural questions about representation also persist, especially as to which representations are being counted and why, and how are probabilistic estimates of being in a parse given the input are formed. The origin of these representations is also unclear, as is the mechanism that is acquiring the statistics and the mechanism that is re-ranking the distributions. If the claim of EBP is that ranking of probabilistic representations what parsing difficulty is, it begs questions as to how the system parses sequences that it has never encountered before, or how it can parse something that is highly unexpected at all, and moreover, what parsing is qua mechanism. If experience is the basis of obtaining probabilistic estimates of a given structural configuration, then it is unclear how parsing might occur without lots of or sufficient experience. Furthermore, how the system acquires experience about parsing, if experience is what is used to generate representations of the parse and probabilistic estimate regarding it, might lead to a circular explanation.

For these reasons, I see the core principles of interference and representing uncertainty as being valuable terms in a larger mechanistic process model, which, hopefully, can also be grounded in neurophysiological computation. By synthesizing mechanistic and Bayesian approaches, we can pose questions both about how language processing functions and why it is that way. But that does not mean that mapping hypotheses about representations and processes onto hypotheses about their priors is will be straightforward.

### Ideal observer models in perception

Ideal observer models have dominated research on perception because they lay bare the computational and statistical structure of the complex problems that the brain solves. They force the researcher to define the information available to the brain, and to construct a quantitative, predictive account of performance (Gibson, 1966; Marr, 1982). The *ideal observer* formally describes human behavior in terms of optimal performance on a given problem or task given uncertainty stemming from the environment or sensory system (Trommershauser et al., 2011). The main source of uncertainty in ideal observer models of visual perception is the probabilistic relationship between a given cue (e.g., contrast, color, shading) and a stimulus (e.g., an edge or object) in the environment. In other words, uncertainty stems from the probability of detection of the stimulus in the face of sensory or neuronal noise (Fetsch et al., 2013). Past experience weights the likelihood function of a cue. Thus Bayesian models that incorporate the right combination of cues and priors have become the best predictors of performance on motor control and visual or multisensory perception tasks (Griffiths et al., 2012; Ma, 2012), although some argue that they need not be Bayesian nor rational to achieve this (Maloney and Zhang, 2010; Rehder, 2011). The psychological mechanism by which the statistical relationship between the state of the environment and internal representation is achieved is not the primary focus of these models, rather finding the formal expression of the statistical relationship between cues, uncertainty, and stimulus such that human behavior is accurately predicted. Once the “right” statistical relationship is uncovered, conclusions can be drawn about the algorithm that best reflects that relationship, and inference can be made as to whether that is indeed what the brain is doing (Griffiths et al., 2012). This approach implicitly assumes that performance or information is optimized, which, of course, does not have to be the case—in fact, a case can be made that energy efficiency or processing time, not information, are what cognitive systems optimize (Friston, 2010; Markman and Otto, 2011).

In any case, ideal observer models have not been prominent in comprehension and production apart from models of reading, speech perception, and rule learning in language (cf. Legge et al., 1997; Norris, 2006, 2013; Goldwater et al., 2009; Frank et al., 2010; Toscano and McMurray, 2010; McMurray and Jongman, 2011; Norris and Kinoshita, 2012). The paucity of ideal observer models in sentence parsing is particularly striking given that we arguably might know more about the formal descriptions of the representations being processed during language use (i.e., formal linguistic representations, perhaps especially during speech perception) than we do about the formal descriptions of levels of representations for visual objects and scenes, or multi-modal sensory representations. One reason ideal observer models might not have taken theoretical hold in parsing, apart of EBP, might be the difficulty in constraining or separating the likelihoods of language processing outcomes that are embedded in the perceptual tasks (button pressing, reading, and making overt linguistic judgments) that most psycholinguistic studies employ. Differences in the task demands of these paradigms may mask, or at least mix in non-straightforward ways, with reliably estimating “pure” language processing likelihoods. Moreover, the source of priors and how they are acquired and updated remains unknown. However, core principles from ideal observer models of perception, namely that including estimates of uncertainty can expose the nature of the problem the brain solves, may be suitable for the addressing the computational challenges that language processing presents.

### Cue combination and integration

**Figure 1:**
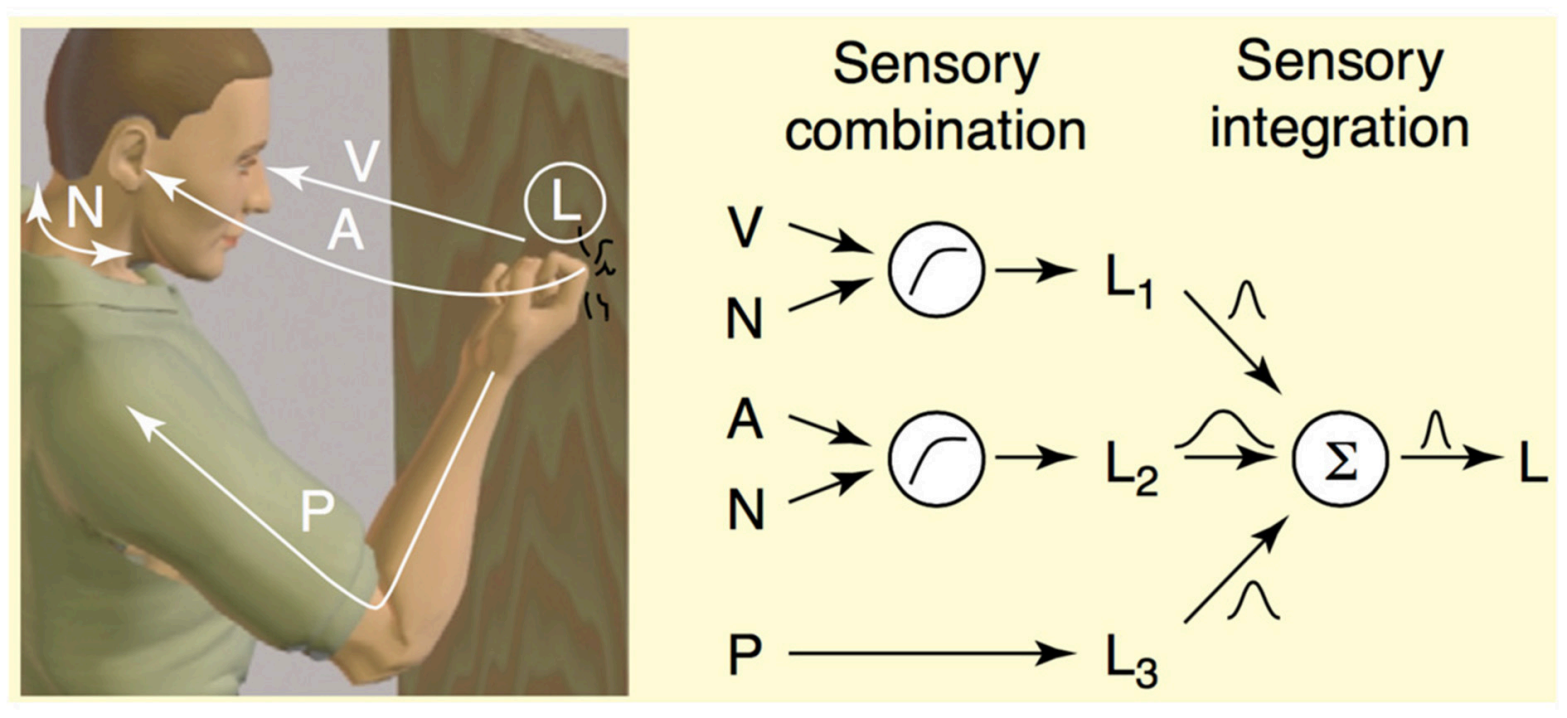
**Illustration of cue combination and integration of the perception during knocking on wood from Ernst and Bülthoff (2004)**. Sensory cue combination occurs between sensory signals that are not redundant, which can be represented in different units or coordinate systems, and which might reflect complementary aspects of the environment, for example visual or auditory information (Ernst and Bülthoff, 2004). This figure from Ernst and Bülthoff (2004) depicts how three sensory estimates about the location (L) of the knocking event are combined to form a stable percept. Information from visual (V), auditory (A), and proprioceptive (P) sensory percepts comprise three different signals about location. Before these signals can be integrated, V and A signals can be combined with the proprioceptive signals (N) to be transformed into body-centric coordinates with the same units. Following that, the three signals (L_1_, L_2_, L_3_) are integrated with their reliabilities to form a coherent percept of the location of the knocking event. Sensory cue integration occurs between so-called redundant signals, or signals that are in the same units or coordinates and that reflect the status of the same aspect of the stimulus in the environment.

In both psychophysical and neurobiological models of perception, *cues* are any signal or piece of information that reflect the state of the environment (Fetsch et al., 2013). For example, when perceiving and localizing an object to act on, such as trying to catch a toddler who is screaming while running away from you, one cue is likely the visual contrast information created by the toddler moving across the visual scene, and another is the screaming, or more accurately the change in interaural time of the screams as the toddler moves in relation to your ears. And lastly, cues can come from any proprioceptive or tactile stimulation that is generated as you prepare to grab your toddler before s/he runs into traffic. Our brains combine and integrate these cues, often from different modalities, to form a stable percept upon which to act (see Figure 1, Ernst and Bülthoff, 2004). The key to stable and robust perception given sampling uncertainty is the integration of multiple sources of sensory information via two important psychophysiological operations, cue combination and cue integration. *Cue combination* is the process of combining cues via summation, and describes interactions between cues that are not redundant in the information they carry. Cues may be in different units during combination, and may signal complementary aspects of the same environmental property. For example, when knocking on a door, one perceives the knock as emanating from the location where one knocked. This percept is the result of the combination of sensory signals from vision, audition, and proprioception (see Figure 1). After cue combination, comes integration, or the weighting of the cues by estimates of their reliability as cue to the true stimulus. *Cue integration* describes an interaction between cues of the same units that may carry redundant signals, and that regard the same aspect of the environment. Evidence across different domains and species implicate cue integration as the mechanism from which stable percepts emerge (Deneve et al., 2001; Ernst and Bülthoff, 2004; Fetsch et al., 2013). Summation is the canonical neural computation, and Carandini and Heeger (2012) argue that normalization, the principle operation underlying cue integration, is also a canonical population-level neural computation for brains of all levels of complexity.

Cue integration is typically expressed in an estimate of the likelihood of the stimulus being present in the environment (Ŝ) given the cues^3^ (*c*_1_…*c*_*n*_*)* and scaled by the reliability of those cues (${\hat{r}}_{1}$…${\hat{r}}_{n}$):

$$\hat{S}=\sum_{i\;=\;1}^{n}c_{i}{\hat{r}}_{i}+\ldots c_{n}{\hat{r}}_{n},\;\hat{r}=\frac{1}{\sigma_{c}^{2}}$$

Equation (1) From Ernst and Bülthoff (2004) the equation above describes the processing moment at the onset of a stimulus. It describes the activation state of a neural population that codes for a given sensory representation. This representation can be said to emerge from integrated sensory cues.

An estimate of *cue reliability* ($\hat{r}$) is the inverse variance of the distribution of inferences made based on a given cue (Bülthoff and Yuille, 1996; Jacobs, 2002). The smaller the variance in the relationship between cue and stimulus, the more reliable the cue is. Correlation between cues also affects their reliability: a cue is regarded as more reliable if the inferences based on it are consistent with the inferences based on other cues in the environment (Averbeck et al., 2006). If a cue is inconsistent with other cues, it is regarded unreliable. Studies on cue reliability have shown that cues that have not changed their value in the recent past are weighted more strongly (Jacobs, 2002). Thus, returning to our example of the screaming toddler, cue combination summates activation from the sensory populations associated with the visual, auditory, proprioceptive, and tactile stimuli that issue from chasing a screaming toddler. Upstream from these primary sensory cue population codes, other neural populations code for combined or composite representations of these cues. At each stage of representation, cue integration weights the representation by that cue's reliability. The reliability of combined cues is equal to the sum of the individual cue reliabilities, so the only neurophysiological operations required are summation and normalization (Fetsch et al., 2012). I will discuss the appeal of this point in Section A Neurophysiologically Inspired Mechanism for Neurobiological Models of Language. Whether cue reliability is best thought of as a prior in a Bayesian framework, or as a probabilistic variable in a Statistical Decision Theory framework is an open question (Maloney and Zhang, 2010; Rehder, 2011). In any case, even non-optimal weighting by cue reliability is probably a better estimate than an individual trial data or single sample measurements (Ernst and Bülthoff, 2004).

## Capturing multiple distinctions in parsing

A desideratum of psycholinguistic theory is a taxonomy of the mental representations and computational mechanisms that language use requires. A particularly satisfying theory would unify the mechanisms occurring during diverse computations such as speech perception, word recognition, parsing into phrase structures, establishing referential and agreement relations, forming long-distance dependencies, and forming discourse representations. Such a theory would have general principles derived from domain general canonical neural computations, and would hold for both for comprehension and production. Processing difficulty would be predictable from first principles, that is, from how the representations at stake are generated. Traditionally, mechanistic theories of language comprehension and production have proposed multiple language-specific mechanisms, often operating at distinct levels of linguistic representation. These have been as diverse as lexical access, reanalysis, binding, lemma selection, and unification (Frazier and Fodor, 1978; Marslen-Wilson and Welsh, 1978; Swinney, 1979; Clifton and Frazier, 1989; Ferreira and Henderson, 1991; Levelt, 1999; Hagoort, 2005), or have invoked heuristics like Minimal Attachment, Late Closure, the Active-filler Strategy, Attach Anyway (Frazier and Rayner, 1982; Frazier and Clifton, 1996; Fodor and Inoue, 1998). Other impactful approaches to parsing have focused on metrics to quantify the difficulty of certain structural configurations in terms of capacity limits on memory, or the number of dependencies to be resolved, or the number of parses to be considered, but not on mechanism *per se* (Just and Carpenter, 1992; Gibson, 2000; Vosse and Kempen, 2000). Yet other dynamical systems approaches to parsing derive empirical phenomena, such as *local coherence*, where local match between constituents' features can override the global parse from architectural aspects of the model (Tabor et al., 2004; Tabor and Hutchins, 2004). A notable antecedent psycholinguistic theory based on cues, albeit with a different goal and level of analysis, comes from Bates and MacWhinney (1987)'s Competition Model (CM), a lexicalist framework focused on the acquisition of grammar in the face of the challenge of cross-linguistic variation. As its name suggests, its main processing claim is that lexical representations compete with each another for case and thematic role assignment during comprehension, and that languages differ in how information is expressed via cues. The CM is an important antecedent for cue integration because it invokes both the notions of cues and cue reliability, but in different senses than in the perceptual literature and thus, than herein. It posits that languages vary in how their forms cue meaning, and in how linguistic form and function are related by cues, and is largely concerned with how different linguistic representation types cue argument relations in different languages and how cues and their reliability facilitate language acquisition. However, the framework I will outline draws strongly on the notion of cues and their reliabilities as internal representations, processed by a neurophysiologically plausible mechanism, rather than on cross-linguistic variation in how information is carved up to cue between form and meaning.

In some ways, mechanistic approaches are just as vulnerable to the criticism of falsifiability that Bayesian approaches are—just as you can change the priors to fit your data—you can, similarly, change the number of hypothesized mechanisms at stake, fail to generate falsifiable hypotheses or testable predictions, or arbitrarily change the architectural bottlenecks in your process model to account for your data (Bowers and Davis, 2012; Griffiths et al., 2012). How does one keep from “over fitting” a process model? Moreover, the frameworks that developed past hypothesized language-specific mechanisms were steeped in the modularity debate, which naturally focused on questions about what operations are language specific or not (Fodor, 1983), and whether processes operated in serial or in parallel (Frazier and Clifton, 1996). Though there is less worry now about sterility and modularity of linguistic representation, and more about incrementally in language processing, it remains a fact that the brain can be said to be modular in its organization (Carandini and Heeger, 2012; cf. Fedorenko et al., 2012) though likely with interesting and important overlap or redundancy in coding in diverse systems (e.g., Schneidman et al., 2003; Puchalla et al., 2005; Rothschild et al., 2010). This presents our desired linking hypothesis between psycholinguistic and neurobiological theories with a conundrum wrapped in a mystery: capturing the incrementally of language processing within a modular system of neural populations, whose coding we do not yet know how to read. In other domains of cognition focused population codes, the relevant questions become: what factors determine the organization of neural populations, what are populations coding for, and how are those representations transformed from population to population (see Pouget et al., 2000; Averbeck et al., 2006)? Translating these questions to a psycholinguistic level of analysis, we then must ask whether signals in brain or behavior that reflect representation of linguistic units can be detected, whether such a modular neural architecture can indeed capture important distinctions for linguistic representation and processing, and whether cue combination and integration alone can account for language processing from speech and visual onset all the way to higher level meaning.

### Language comprehension as cue combination and integration

Can a satisfying analogy can be made between language comprehension and perceiving a complex natural environment? Like object perception or localization, scene perception, or motor control, language processing is multimodal. In conversation, language comprehension minimally involves integration of auditory and visual information^4^. All this must occur while planning and producing language in return. Furthermore, language use is highly goal-directed and joint, an issue that is rapidly gaining theoretical importance (Pickering and Garrod, 2004; Gambi and Pickering, 2013; MacDonald, 2013). But aside from the issues of modality and joint-action, language may present a processing situation that fundamentally differs in the kind of representational relationships that the brain must form in order to explain linguistic taxonomy. Information from multiple, sometimes hierarchical, sources of formally discriminable representations must be perceived from the environment. Extracting linguistic representations from a speech or visual input may be, in some ways, analogous to the binding problem in vision and attention (cf. Treisman, 1999). In both situations, information that is distributed over time and space at different frequencies must be grouped or bound into higher-level representations for processing to occur. Cues, whatever they may be, from each sensory input level are combined and integrated with their reliability estimates, and emerge as a linguistic representation, e.g., a phoneme or phrase. Populations coding the reliability of a given representation as a cue to higher-level representations are activated and updated. Those reliabilities are integrated with the population code representation for a given representation, which in turn produces the next level of representation.

**Figure 2:**
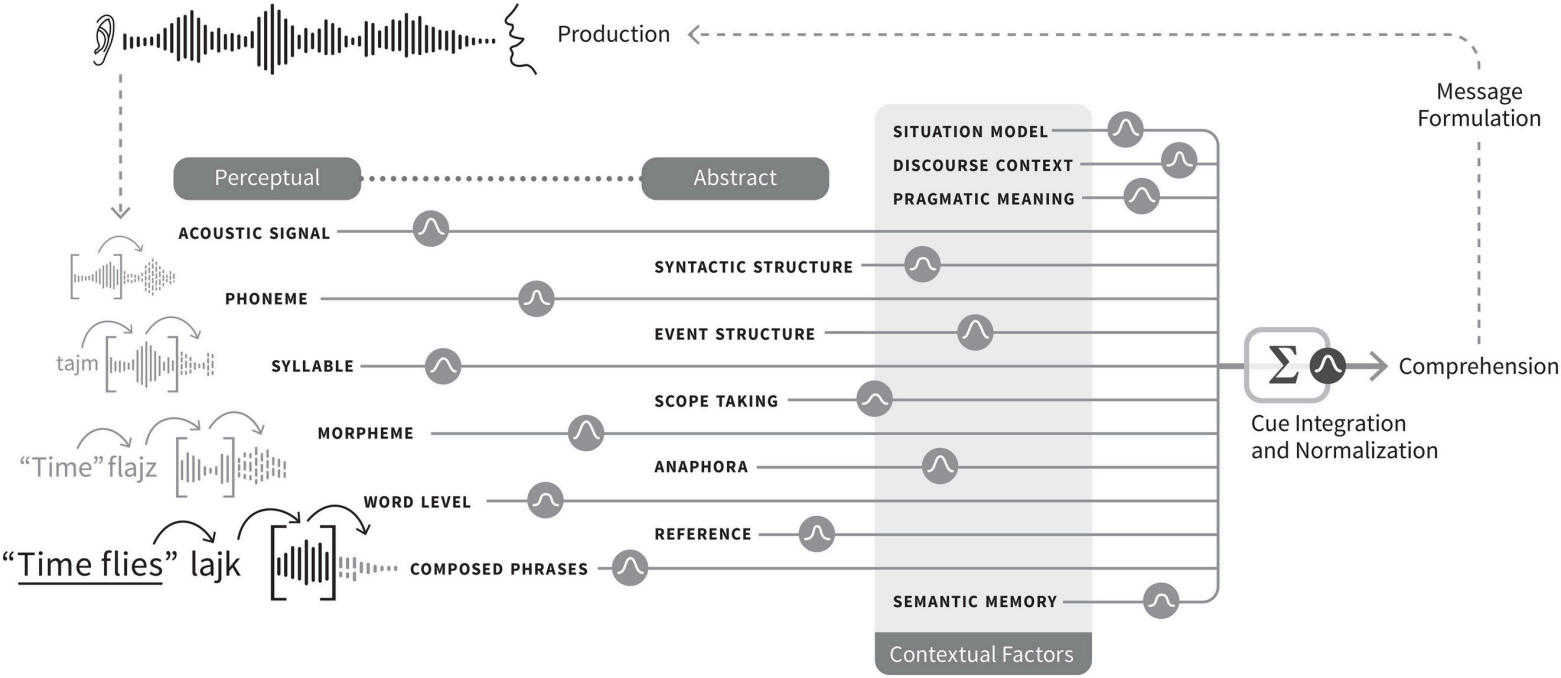
**Graphical representation of the cue integration architecture of representation and processing**. Gaussian icons represent integration of cues and reliabilities. Each text label corresponds to a neural population coding the representation. Each text label has a corresponding estimate of *S* from Equation (2), which describes the activation of the population represented by the shape. Each transition between levels of representation, denoted by the Gaussian icons, can be described formally by estimates of *r* and *l*, or cue reliabilities. Activation can spread such that cueing of the next representation occurs before processing of the current set of features completes, such that emerging representations can serve as cues to related representations, where a word or phrase level representation can receive stimulation from a morphemic or syllabic or prosodic representation, and vice versa. This aspect is visualized by both the lines terminating in the summation symbol and by the fact that the individual text labels are not connected to one another. The lines or pathways of processing terminating in the summation box are meant to illustrate that information represented at these levels of the model can be of multiple, co-extended levels of linguistic representation, and perhaps also redundantly coded. This redundancy might also hold at phrasal levels and above. Similarly, such a box model could be represented across dual streams in a neurobiological model where one stream carries out sequential processing. If so, then aspects of representation that are order invariant would be represented in one stream, and order-sensitive aspects in the other, resulting in another form of redundancy. Predictive coding is represented by the small arrows passing information forward in time to bias the processing incoming input. Figure design and implementation by Erik Jacobsen of Threestory Studio.

As in the psychophysical literature, most of the explanatory work would be carried out by cues, a notion that is difficult to define both in the positive (what cues are), and in the negative (what can't be a cue). In fact, often the term “cue” is treated as if should be implicitly understood, as in, as if it has no specialist or jargon meaning. In the perception literature, a cue is any sensory information that gives rise to an estimate of the state of the environment (Ernst and Bülthoff, 2004). Here I will augment that definition as follows: a psycholinguistic cue is any internal representation that signals, indicates, or is statistically related to the state of some property of the environment relevant for language processing. Thus, a cue to a given psycholinguistic representation is simply any representation that is reliably related to that given representation, in contrast with a representation that is not related to it. The only way for this simple definition of cue to become explanatory is if it can speak to how abstract linguistic representations might be formed from perceptual inputs, or more specifically, formed from an interaction or convolution of sensory percepts with extant knowledge (read: other representations) in the brain^5^. The problem of satisfactorily defining a cue for functional use in a process model bumps up against the even harder problem of defining mental representation, or defining what perceptual or cognitive features are. Both of these philosophical challenges are, luckily, beyond the scope of this model. However, the functional role of cues may be to simply to map out the structure, path, or links between representations as they are activated in moment-to-moment processing. In this sense, is it not so much what cues precisely are that matters (although that is no doubt an important, troubling question), but which representations cue which other representations to form a map of language processing, from percept to abstract representation that matters for a model. Thus, cues are representations of linguistic input *and* what links those representations in a “chain” for processing from sensory input to abstract representations. I will sketch how a cue integration model might handle processing from speech onset to phrase or sentence comprehension (see Figure 2 for visual illustration). I simplify the representational levels at stake as: phonemes, syllables, morphemes, words, phrases, syntactic and event structures, and discourse context.

### Sensory resampling to recover hierarchical representations

In the case of linguistic representations, aside from the first perceptual cues to enter the processing stream, further cues must come from the same sensory input: a sort of resampling of the sensory percept, or a form of perceptual inferencing (Ernst and Bülthoff, 2004). This resampling would recover hierarchical representations in memory that are activated by that percept, via the same cue integration mechanism that is hypothesized to work for exogenous cues. In other words, cue integration can take as its input an endogenously stimulated representation or set of sensory features (e.g., phonemes from acoustic features, or on a higher level, morphemes and lexical entries), and output another pattern of activation or representational state (e.g., syllables from phonemes, or on a higher level, phrases). The representations from the last cycle of processing serve as cues to the next level of representation or cycle of processing.

The architectural hypothesis is that each level of representation is a cue to higher levels of representation, resulting in a cascaded architecture: phonetic features are cues to phonemes that are cues to syllabic and morphemic representations, which in turn are cues to lexical and phrasal representations, leading to phrase-based parsing and larger sentential or event structures. Figure 2 illustrates how the phrase *Times flies like an arrow* would be processed using cue integration. Activation can spread such that cueing of the next representation occurs before processing of the current set of features completes, such that emerging representations can serve as cues to related representations, where a word or phrase level representation can receive stimulation from a morphemic or syllabic or prosodic representation, and vice versa^6^ (see Figure 2 for illustration). As the phonemes in *time* are parsed, they cue the morpheme and word representations of “time,” which in turn activates syntactic or structural representations, and conceptual representations associated with *time* (e.g., phonotactically licensed syllables, verbs, phrases, related semantic knowledge).

Population coding parameters would constrain how information is represented in the model, but how can such a radically interactive and redundantly coded system be represented? An efficient way to represent a true multitude of representations without overcommitting neural “real estate” might be opponent channel processing. In color vision, a multitude of colors are perceived from photons interacting with photopsin proteins that are tuned to different frequency spectra in cone cells in the retina. The activation patterns of these cells together form opponent channels, where a given channel can be said to detect the difference in activation between cone cells (with different photopsin proteins) tuned to two opponent ends of a spectrum of light (e.g. red and green, blue and yellow), rather than representation via a series of cells or ensembles dedicated to each color or frequency band. Such an opponent system has also been implicated for spatial coding in auditory cortex, where, while most auditory neurons respond maximally to sounds located to the far left or right side, few appear to be tuned to the frontal midline (Stecker et al., 2005). Paradoxically, psychophysical performance reflected optimal acuity in the frontal midline, thus the existence of an opponent process system synthesized these apparently conflicting findings (Stecker et al., 2005). Opponent processing may be a possible architectural feature to represent a multitude, or even a discrete infinity, of linguistic representations via cue integration (e.g., of minimal pairs or other representations in complementary distribution), though it thus far observed has only been observed in much more primary or lower-level sensory processing stages. An opponent channel representational system, operated on by cue combination and integration, would likely be able to flexibly and efficiently code the number of representations needed for such a massively interactive architecture without taking up an implausible amount of neural real estate.

While cues determine which representation is activated, cue reliabilities determine the strength of the evidence for a particular representation and thus how good of a model of the world the system has. To create and maintain an accurate and robust set of representations reflecting the linguistic environment, reliabilities need to reflect local context as well as latent knowledge, or a global prior. Cue integration can account for processing variables in one of two ways: either by modulating the information expressed in the cue reliabilities, or by modulating the circuit of representations, the order or domain of cue computations. Information from memory might be expressed as both an immediate prior (*r*), representing recent processing and the local environment, similar to the notion put forth by Jaeger and Snider (2013), and as a more stable, long-term set of global priors (*l*) that reflect information like discourse context and pragmatic meaning, and semantic and world knowledge. In the set of expressions below, I separate reliability into two terms (see Equation 2). Although both terms are subject to summation, I want to make it clear that they represent different sources of uncertainty, that are likely to be represented by different populations, or redundantly on different levels.

$$\hat{S}=\sum_{i\;=\;1}^{n}c_{i}{\hat{r}}_{i}{\hat{l}}_{i}+\ldots c_{n}{\hat{r}}_{n}{\hat{l}}_{n},\;\hat{r}=\frac{1}{\sigma_{ci}^{2}},\;\hat{l}={\sum_{i\;=\;1}^{n}\frac{1}{\sigma_{c}^{2}}}_{i}$$

Equation (2) Ernst and Bülthoff (2004)'s expression of likelihood of activation adapted to parsing. It describes the activation state of a neural population that codes for a given representation. This estimate of activation is composed of cues (e.g., representational features or any representation), weighted by their reliability, or the likelihood that the stimulus is in the environment given the cue. Estimate of *S* is the likelihood a level of representation is activated by the cues or representational features denoted by *c*, weighted by their reliability *r*, the recent inverse variance of the link between that cue and its related or antecedent representation, and by its latent reliability *l*, the global reliability of that cue over a longer time scale. Estimates of *S* would describe the activation represented by any of the shapes denoted in Figure 2, while estimates of *r* and *l* would be denoted by the arrows feeding forward on back between each level of representation.

To unpack the cue integration process, we can take the example phrase “Times flies like an arrow…” from Figure 2, and examine how the first two words *time* and *flies* would be extracted from the phonemic stage to achieve the morphemic- lexical stage. I will outline how Equation (2) would describe this step in processing. The phonemic string /tajmflajz/ has been parsed from acoustic information^7^, so the next step is for /tajmflajz/ to cue the morphemes/words [tajm|*time*] and [flajz|*flies*] into the phrase *time flies:*

Cartoon process: */tajmflajz/-*>*[taj][m][flajz]-*> *time, flies -*> *Time flies*

$$\begin{array}{l} \hat{S\left[\left[tajm\right]\right]}=\sum_{i=1}^{n}c_{\left[taj\right]}{\hat{r}}_{\left[taj\right]}{\hat{l}}_{\left[taj\right]}+\ldots c_{\left[m\right]}{\hat{r}}_{\left[m\right]}{\hat{l}}_{\left[m\right]},\\ \hat{r}=\frac{1}{\sigma_{ci}^{2}},\;\hat{l}=\sum_{i\;=\;1}^{n}{\frac{1}{\sigma_{c}^{2}}}_{i}\\ \hat{S\left[\left[time\right]\right]}=\sum_{i=1}^{n}c_{\left[tajm\right]}{\hat{r}}_{\left[tajm\right]}{\hat{l}}_{\left[tajm\right]},\;\hat{S\left[\left[flies\right]\right]}\\ \;=\sum_{i=1}^{n}c_{\left[flajz\right]}{\hat{r}}_{\left[flajz\right]}{\hat{l}}_{\left[flajz\right]},\\ \hat{S\left[\left[time\;flies\right]\right]}=\sum_{i=1}^{n}c_{\left[tajm\right]}{\hat{r}}_{\left[tajm\right]}{\hat{l}}_{\left[tajm\right]}+c_{\left[flajz\right]}{\hat{r}}_{\left[flajz\right]}{\hat{l}}_{\left[flajz\right]},\\ \hat{r}=\frac{1}{\sigma_{ci}^{2}},\;\hat{l}=\sum_{i\;=\;1}^{n}{\frac{1}{\sigma_{c}^{2}}}_{i} \end{array}$$

Equation (3) Describing processing moments from the phonemic representation of /tajmflajz/ cueing the words *time* and *flies*, and finally the phras*e Time flies*

We can already see that the description of the activation of the model or system as described by Equation (3) is completely dependent upon the time step or processing moment that we choose to analyse or observe. The importance of time step may not be an issue for implementing a computational model based on cue integration that is dynamic in its activation, but it certainly is a theoretically troubling issue. Would processing moments or cycles be determined solely by the external stimulus, e.g., by speech envelope? Or would the current state of the system upon input instead structure processing time, for example, actually result convolving current activity with the incoming physical (and later, the abstract linguistic) properties of the input? I will explore this problem more in Section A Neurophysiologically Inspired Mechanism for Neurobiological Models of Language.

A second important consequence of cue integration is that it implies a hybridized notion of modularity: perceptual representations might still be encapsulated in the Fodorian sense, but once representations become either multi-modal, or are resampled as the cue other representations further up the processing stream, they are no longer so. In fact, as a reviewer pointed out, higher levels of representation in such a model would flatly deny Fodorian modularity (see Fodor, 1983). Another way of putting it is that, under cue integration, early representations, which tend to be perceptual, may be encapsulated until they are summated with other cues. This hybridized modularity would also play out in terms of deeming the pathways and networks that process the representations to be domain-specific or not.

Returning to the important psycholinguistic notions captured by EBP and CBR, how would a cue integration model cache out surprisal and interference? Surprisal might be cached out in terms of sub-optimal cue integration with reliability, poor trading off of global cue reliabilities for recent ones, such that global reliabilities are overweighting the current representation. Interference would amount to sub-optimal cue combination, where the cues for a competing parse or related representation activate an “attractor” representation, instead of the true stimulus. It would arise when sub-threshold activation is shared between representations that share features with the input, a form of cue overload, and may or may not fully activate the “attractor” representation. Cue overload in such a system would still depend upon how diagnostic a cue, or summated cue set, is to a unique representation in the system. Garden-path effects and other parsing ambiguities might be cached out in terms of poor estimates of recent reliabilities compared to global ones, such that summated cues point to ultimately ungrammatical representations. A cue integration process model would extend the notion of cue combinatorics during retrieval and formation of non-adjacent dependencies (Clark and Gronlund, 1996; Lewis and Vasishth, 2005; Van Dyke and McElree, 2011; Kush et al., 2015) to a general processing principle and makes a claim about how cues are combined with one another. The model would assert that processing difficulty is essentially always a form of cue overload, which stems from architectural first principles of how activation of representations occurs and how uncertainty flows through the system dynamically.

Even the first input step in sketching a processing stream is grossly oversimplifying and glossing over important and vibrant subareas, especially in the neurobiology of speech perception (Hickok and Poeppel, 2007; Poeppel, 2014). Recent compelling evidence suggests that neural populations entrain with an auditory stimulus using acoustic-phonetic “sharp edges” to latch onto the speech envelope (Luo and Poeppel, 2007; Doelling et al., 2014; see Poeppel, 2014 for discussion). Giraud and Poeppel (2012) show an emerging role for oscillatory activity as entrainment with speech envelope and syllable structure. This entrainment could be performing cue combination and integration of phonetic features into phonemes, but a clear experimental question is if cues and their reliabilities are coded in or recoverable from oscillatory activity. Such a simple process model must be able, at minimum, to capture the vagaries of speech perception, it being the stage of language processing most firmly grounded in perceptual processing (Samuel, 2001; Samuel and Kraljic, 2009).

### Representations and grammar

An issue that will clearly determine the success of a cue integration process model is the nature of the representations the model posits. The basic representational claim of a cue integration process model is that representational features make up a level of representation, and serve as cues to subsequent levels. They do so in a cascaded way and incorporate at least two error terms. This would mean that the system's organization comes from, or even just *is* the grammar of the language it was trained on. But probably any cue-based model also makes that claim that ungrammatical representations might be formed if the rest of the cues, i.e., non-structural ones, point toward a given representation. One way to avoid the “bag of words” problem (Harris, 1954), where semantic and other non-structural features dominate over structural relations would be to simply weight syntactic features more strongly in their reliability.

Without a traditional mechanistic structure that assumes multiple operations, one possible consequence is that representations need to be similar to something like slash categories in a combinatorial constituent grammar, as in Combinatorial Categorical Grammar (CCG; Szabolcsi, 1989, 2003; Steedman, 1996, 2000; Jacobson, 1999). If they were, then the dependencies that are cached out as empty categories in other grammars, as well as other forms of dependency, could be carried forward during processing without the need for positing constructs like buffers or maintenance^8^, because the dependency is represented as a grammatical feature that can “percolate^9^” to the highest tree, representation or population code. Separate operations for retrieval and interpretation may also become moot if grammatical features (of which dependency is now just one example of) can percolate up the path of population codes. By caching out problems like non-adjacent dependency as representational feature parsing, CCG, and perhaps cue integration, perform the classic programmer's trick of changing data structures to increase expressive power when of the processing architecture. However, this trick only means that the difficulty is merely transmogrified—now the cue integration process model is generating hypotheses about both psychological processing mechanisms *and* about the nature of representation. This is especially problematic because traditional dependent measures (e.g., performance on a task, brain responses, but especially reaction times) cannot discriminate between effects arising from differences in processing speed (a proxy for mechanism) and differences in representation strength or other aspect (Wickelgren, 1977; Davidson and Martin, 2013). This means that experimental designs will have to be careful not to conflate predictions about representation with predictions about mechanism itself. The speed-accuracy trade off procedure (SAT; Reed, 1973) offers a way to measure effects of processing speed orthogonally from representation-based differences, but it relies on an overt metalinguistic judgment. Given cue integration's grounding in perception, it is not unreasonable to think that SAT could be applied to study both the representations of cues and their reliabilities, especially because discriminability between signal and noise, or *d'*, is composed of hits (yes responses to trials from the signal distribution) and false alarms (yes responses to trials from the noise distribution). Nonetheless, deriving testable predictions about the natures of the representational architecture in a cue integration process model for behavioral data will be challenging.

## A neurophysiologically inspired mechanism for neurobiological models of language

How can we formulate a meaningful linking hypothesis between a psycholinguistic process model and current circuit-based neurobiological theories of language? First we must try to formulate it in term of mechanisms that are both grounded in canonical neurophysiological computation and psychologically meaningful. The class of neurobiological models exemplified by Hickok and Poeppel (2004, 2007) focus on sub-lexical processing and speech as the first information-processing hurdle. Such models tend to have more fine-grained, detailed claims about neurobiological architecture than models that focus on syntactic or semantic processing (Hagoort, 2005, 2013; Friederici, 2012), although some very recent phrase and sentence level models are becoming much more articulated in the complexity of the dual-stream circuitry and in claims about directionality and interaction of processing streams (Rauschecker, 2012; Hagoort and Indefrey, 2014; Bornkessel-schlesewsky et al., 2015; Friederici and Singer, 2015). In any case, trying to find a mechanistic foothold can be difficult. Cue combination and integration maps broadly onto the general concept of Unification from Hagoort (2005)'s Memory Unification and Control model, as a mechanism to combine processing units into larger, hierarchical structures. In MUC, unification is separated by modality or representational type, such that phonological, syntactic and semantic unification are separate, as are the processing streams that deal with them (Hagoort and Indefrey, 2014). A cue integration model would not stipulate encapsulation by formal representation class but, rather, by order of cue summation and thereby connectivity of the populations, which may or may not turn out not to be equivalent to representation class.

The cue integration model also differs from Unification in that it makes the claim that uncertainty, specifically cue reliability, is integrated with the population activation for a given cue or cue set. This would mean that cue reliabilities would need to be dynamically updated, and more broadly, that the representations carried by a given neural circuit would need some element of flexibility and would be robust due to redundant coding of features across certain populations. They would also need to be robust, and so redundantly represented in multiple populations. Friederici and Singer (2015) propose that the sparse, flexible, feature-based coding that is seen in other cognitive systems applies to linguistic representations in the brain. In such a system, there is both temporary coupling of populations coding cues or features of larger representations, as well as lasting couplings or “firmware” of anatomical assemblies, as outlined in Singer (2013). Careful experimental work would be needed to test this hypothesis and to determine if flexible sparse coding can handle formally complex linguistic representation, and furthermore, to determine which aspects of phonological, lexical, syntactic, semantic, discourse, or pragmatic representations are flexibly coded or “hard coded.” Such an architecture would be highly suited to a cue integration process model but in combination with redundancy in coding to generate robust representations. Such an architecture may enable the system to represent discrete infinity.

To emphasize, the only computational mechanisms stipulated in a cue integration process model would be *summation*, the neurophysiological mechanism for cue combination, and *normalization*, the neurophysiological mechanism that integrates a cue with its reliability. If parsing and other language processing phenomena can be accounted for using only these two stalwart neurobiological mechanisms, it would be a step in the direction toward a unified theory of human information processing that includes language but is based on “brain-general” processes.

### Cue integration and forward models

Another powerful capacity that any process model would need to account for is the role of predictive processing in language behavior. Forward models from vision and motor control have already had some influence on theoretical work in cognition and language (Pickering and Garrod, 2007; Pickering and Clark, 2014), but have yet to be fully specified in models with clear predictions for language processing. In a classic computational model of vision, Rao and Ballard (1999) describe an architecture wherein top-down feedback connections carry predictions about bottom-up or lower-level population codes, and feed-forward connections carry residual error between those top-down predictions and the actual input. They illustrated that in this kind of forward model, architectural facts about the visual system, such as receptive field characteristics and surround suppression^10^, emerge naturally. This seems to suggest that such architectural features occur as a result of cortico-cortical feedback, and that cortico-cortical feedback is a promising candidate mechanism for predictive coding (Rao and Ballard, 1999). Synthesizing predictive coding via cortico-cortical feedback with a cue integration process model, feed-forward connections would carry bottom-up activity corresponding to integrated cues and reliabilities. A subset of feed-forward cue reliability activity would be the error signal in response to predictive activation forecast via the top-down feedback circuit. Although predictive coding and forward models will no doubt play a larger role in psycholinguistic theory in the coming years, the fact that we can understand the unpredicted or unexpected utterances at all, or with reasonable ease, suggests that prediction is not the core language processing device (see also Jackendoff, 2002; Rabagliati and Bemis, 2013; Huettig, 2015; Huettig and Mani, 2016). But the fact remains that predictive coding plays a huge role in most sensory processing domains, so any model of language ought to have an architecture that can implement it using existing neural infrastructure.

### Cue integration in a neurobiological circuit

A cue integration process model could make contact with neurobiological models in two ways: (1) in terms of the claims being made about the cue-based computations being carried out in various neural circuits, and (2) in terms of the implied population codes or representations needed in a given circuit. The first issue returns to the question of how to falsify hypotheses about the number and kind of processing mechanisms. A way to circumvent the problem is to focus on the end-state computation or the transformation that a representation undergoes in a given processing stream.

In a similar spirit, Bornkessel-schlesewsky et al. (2015) derive a dual-route model for human language processing from speech to syntax that is rooted in primate audition (Rauschecker and Tian, 2000). The key differences between the antero-ventral and postero-dorsal pathways in Bornkessel-schlesewsky et al. (2015) is time invariance or order sensitivity: the antero-ventral stream processes or extracts increasingly complex hierarchical auditory representations with commutative properties whilst the postero-dorsal stream processes sequence information or is order sensitive. The postero-dorsal stream makes use of forward models via an efferent copy that carries predictions and detects error, enabling sequential order-sensitive processing (Bornkessel-schlesewsky et al., 2015). The cue integration process model does not make any claims about the location or make-up of language circuits, nor does it have fundamentally different assumptions about basic representation types (phonetic features, phonemes, lexical, phrasal, event, etc.) that many extant models posit. Rather, cue integration makes a specific claim about (1) the psychological and neurophysiological mechanism underlying formation of these representations (i.e., summation of population codes for cue combination and normalization of those codes for integration with reliability), and (2) the representational infrastructure (e.g., dynamic and redundant population-level encoding of feature-based representations and uncertainty about them).

The debate about the modularity of language from other cognitive systems has featured compelling arguments that theories of language evolution must shape or constrain theories of language and language processing (Hauser et al., 2002). The claim that language evolved too recently to derive a new domain-specific neural mechanism is linked to the notion that brain processes can be repurposed to suit timely organism-environment interaction needs (see Gervain and Mehler, 2010 for discussion; Knops et al., 2009). Cue integration is a good candidate for such a repurposed process. However, though the cue integration architecture can represent recursion in principle, that fact alone cannot explain why recursion is not more widely found in other representational systems in cognition (Jackendoff and Pinker, 2005). That is unsatisfying, especially if, in a hardline reductionist thought experiment, one really wants to claim that there is only one neurophysiological brain process relevant for cognition (or extraction of further representations from sensory input), and that process is cue integration. To entertain such a thought experiment further, or for such a reductionist position to be tenable, language also needs to be learnable using only cue integration over the cue-based architecture with reliabilities.

## Cue integration in language acquisition and bilingualism

A crucial aspect of any theory of language is that it must be learnable. How might representations be acquired under the assumptions of a cue integration process model?

The cue integration model does not radically differ from current thought on language development—it would hypothesize that linguistic representation develops in the infant as a function of perceptual cue decoding via statistical learning (Saffran et al., 1996), but that first hierarchical representations depend on acquiring a cue-based architecture and cue reliabilities, which in turn shape the development of the assembly networks. Much of the same how-why camp tension exists in language acquisition between pure statistical learning-based accounts and nativist process models (Kuhl, 2004; Gervain and Mehler, 2010). Gervain and Mehler (2010) argue that the hard work for language acquisition theorists is discovering how the system combines statistical learning and rule acquisition or language-specific cues. Only from this combination can an account capture cross-linguistic variation and sensitivity to language-specific cues in infants and neonates (Kuhl, 2004; Gervain and Mehler, 2010). To this end, Gervain and Mehler (2010) synthesize nativist and statistical learning accounts of speech processing up to the acquisition of morphology, concluding that some types of linguistic representations may be more suited to statistical learning (e.g., consonants) than others (e.g., vowels). But the challenge lies in how acquisition occurs in learning situations where, for example, frequent monosyllabic speech that arises as in some infant directed speech and even in some languages, which renders statistics like transitional probability useless (Gervain and Mehler, 2010). Under their account, acquiring complex hierarchical representations must capitalize on both the statistical information from the linear of sequences, and on language-specific cues, or the formal representations of a particular language, but how that tradeoff or interaction occurs is of course unknown. The cue integration process model offers an architecture that may be able to capture both statistical learning aspects (via reliabilities) and rule-based aspects (through assemblies or cascaded cues networks). In order to avoid some of the same criticisms lodged earlier in this article, the cue integration model needs to be able to derive abstract hierarchical representations from noisy, sparse inputs with few priors. That seems dubious at the moment, mainly because the representations or the bias toward forming certain types of representations would have to be innate. This situation echoes the learning problem that statistical models usually face: how do you parse input without the representations to do so? In other words, how do you count anything if you don't know what it is you are trying to count? I turn to a model of concept learning for inspiration because learning by analogy seems to avoid many of the pitfalls of both nativist and statistical accounts (Doumas and Hummel, 2005), as well as having some striking computational overlap with current neurobiological models of language.

At least at the level of the sentence, the tension between statistical and nativist perspectives might be eased somewhat by well-articulated claims about acquisition of relational concepts like *above, bigger*, or *more*. The Discovery of Relations by Analogy (DORA) model of relational concept development by Doumas et al. (2008), uses associative learning to create symbolic, hierarchical relational concepts from linear input sequences. DORA learns multiple argument predicates using time or onset of activity in sub-nodes, or systematic synchrony or asynchrony of firing of the sub-nodes representing each argument^11^. In other words, DORA learns *bigger than* (X, Y) by predicating *larger* (X) and *smaller* (Y) and combining these single argument predicates by their occurrence in time, such that the model can discriminate between X is bigger than Y and Y is bigger than X (see Doumas et al., 2008 Figure 3 for illustration). Such a strategy would work well in a redundant, flexible architecture that is also self-organizing and associative in nature (cf., Singer, 2013; Friederici and Singer, 2015). Firing asymmetry offers an additional level of description or representational state for the model without positing another psychological mechanism or neurophysiological process. Modeling, in combination with empirical work, would of course be needed to substantiate any of these claims.

For the bilingual brain, the cue integration model has modest implications but casts several existing questions in relief. First, that reliabilities and cue architecture may or may not be shared between languages (Nieuwland et al., 2012). Second, that age of acquisition might determine how assemblies are formed (Nieuwland et al., 2012). Third, proficiency may be cached out as differences in network density, representational interconnectedness, or unstable reliabilities, all of which could underlie non-native performance for bilinguals. If assemblies are malleable until the critical period is over, at which point only reliabilities are in flux as a function of language experience, any subsequent language learning would require the system to use alternate circuits to form new language-related assemblies, resulting in differing neural infrastructure that can (but does not have to) affect the competence and performance of late bilinguals.

### Cue integration in production and dialogue

Regarding performance, the challenges facing an integrated theory of comprehension and production endure. Questions like whether the same representations are used in comprehension and production or whether analogs or “mirror image” representations are working in concert during production and comprehension are exciting but difficult to test. Brain imaging evidence suggests that similar areas are engaged during production and comprehension (Rauschecker and Scott, 2009; Menenti et al., 2011) but whether the representations at play are identical or analogous is not yet clear. Certainly an important interaction occurs that leads to suppression of activity in auditory cortex in response to one's own speech (Numminen et al., 1999). Cue integration would make a claim about the process through which representations are activated during production, and there is no principled reason why the cue integration process and cue-based architecture cannot be the same in both processes. However, reliabilities pertaining to the representations might need to be different for comprehension and production. Regarding the claim that prediction is based on production (Pickering and Garrod, 2007, 2013) and the claim that production difficulty is at the root of comprehension difficulty (MacDonald, 2013), cue integration forces an opposing view. Cue integration stipulates that the cue-based architecture for language arises from perceptual processing. There are several difficult challenges for the account to claim otherwise: first, if cue integration is a repurposed neurophysiological mechanism from perception, and it gives rise to linguistic representations from auditory percepts, then it is fundamentally based on comprehension, at least during acquisition. Secondly, comprehension occurs before production during development, furthering support for basing at least the origins of linguistic representation in comprehension. Third, receptive vocabulary is larger and accrues faster in development, and is larger in bilinguals (Benedict, 1979; Laufer, 1998), so it is unclear how these facts fit into a model where comprehension and production draw on exactly the same representations. These arguments do not exclude the possibility that a significant portion of cue reliability during comprehension is uncertainty stemming from dynamic production-based experience in the adult, leading to a situation where comprehension difficulty is rooted in a production-based variable, as MacDonald (2013) argues.

Producing an utterance in the cue integration architecture would go as follows: activation for an event structure cascades down representational levels in a planning-cycle-sized chunk. The cue-architecture basically fires in reverse order, and reliabilities include uncertainty from articulatory planning and other production-based priors. Predictive coding would also have to operate in the opposite direction. The system would still be susceptible to cue overload whether or not production and comprehension representations are identical. Coupling between processing streams or analog representations during both production and comprehension could occur.

During dialogue, language production often based on comprehension of what was just by an interlocutor. If production reverses what is top-down and bottom-up and changes the predictive coding direction, then dialogue is a cascaded engagement of this stream coupled with the comprehension stream. In dialogue, these streams become coupled between two brains, forming a sort of ultimate cacophony of synchronous and asynchronous firing. The only new claim a cue integration model would make is that cue reliabilities would then have endogenous and exogenous sources, from the speaker and interlocutor, and would crucially have to contain predictions about the interlocutor's representational states. Alignment then might be cached out as how well-entrained dialogue partners' cue reliabilities for each other are. Cues in dialogue might also place more weight on non-linguistic percepts or cues, which may end up influencing the reliabilities of linguistic representations, for example, gaze, facial expression, gesture, and goal-directed or joint-action contexts and behavior. Turn-taking and other time-based behaviors between interlocutors would be entrained with or based on asynchronous firing across speakers (Stephens et al., 2010).

### Predictions from cue integration and persistent challenges for any cue-based model

The real work for this developing theory is generating testable predictions. What can a simple process model based on psychophysiological principles mean for brain data and for behavior?

Given the architectural nature of the claim, a starting point might be computational models of language that are based on primate and avian auditory processing (à la Doupe and Kuhl, 1999; Rauschecker and Tian, 2000; Bornkessel-schlesewsky et al., 2015) using associationist learning to acquired symbolic representations. If such a computational model can approximate human learning and processing of language, it would still be a form of confirmatory evidence rather than an attempt at falsification. But such an implemented computational model might be able to generate finer grained predictions for electrophysiology and behavior.

Another approach to falsification might be via the manipulation of the cue relationships between representations, and of cue reliabilities, in an artificial language. This approach would try to manipulate the reliability of a phoneme as a cue to a morpheme, or a morpheme as a cue to a phrase structure, to see if participants track reliabilities and if manipulating them affects reading time. Cue integration also predicts that a noise term for each level of representation should exist. An elegant point from Maloney and Zhang (2010) is that one way to falsify Bayesian accounts it to observe that estimates of priors transfer onto other trials or related tasks. Thus, estimates of priors might be expected to transfer onto other item sets, syntactic structures, lexical items, discourse or information structures. It is yet unknown how much of a role individual differences in language experience might contribute to both recent and global priors or cue reliabilities.

Another class of predictions the cue integration model might make regard neuroimaging data. Although the relationship between something like a population code and an electrophysiological frequency band or event-related component is highly speculative at best, I will try to generate predictions both on the population level (though they are not yet measureable in humans apart from intracranial electrocorticography), and try to predict an analog for a signal our existing psycholinguistic electrophysiological dependent measures can detect. First, formal linguistic distinctions in a particular language should determine population codes. Under an opponent processing system, the opponents in a channel would be determined by that language's minimal pairs at various levels of representation. Beyond the population level, such a language-specific population coding architecture's first fundamental prediction is, certainly for abstract constructs like event-related brain potentials (ERPs), for variety of indices (i.e., different ERP components elicited by strings with the same meaning across languages) showing sensitivity to different processing variables across languages (see Bornkessel-Schlesewsky et al., 2011).

Second, if firing asynchrony is important for perceptual grouping (both in processing and in learning), then a cue integration approach predicts a lack of phase in electrophysiological signal with stimulus onset. This “delay” should be true for population codes, oscillatory activity, and ERPs. But there should be some temporal relationship with onset as a function of the number or complexity of representations being extracted from the auditory percept (Luo and Poeppel, 2007; Giraud and Poeppel, 2012; Golumbic et al., 2013), though discovering what that relationship seems very challenging. Nonetheless, discovering the relationship may make contact with neurophysiological principles about oscillatory activity, namely regarding questions as to how oscillatory activity is driven both by the temporal properties of the incoming, exogenous stimulus *and* by the current endogenous processing moment, and what the nature of the relationship between those two oscillation timescales is. Third, the cue integration model, which is built on cue reliabilities, or the representations of the probabilistic relationship between a given cue and an upcoming representation, predicts that there should be some neural signal that is related to the reliability of each level of representation as a cue to the next.

At least two fundamental problems seem to endure for a cue-based model. First, a persistent challenge is understanding why processing similar representations before or after the onset of a target representation is sometimes facilitatory (resulting in priming) and at other times inhibitory (resulting in interference). Is firing asynchrony somehow underlying the spectrum of priming and interference? Second, how might long-distance structural relationships, syntactic domains, and scope be encoded in a cue-based direct-access system (see Kush, 2013 for a discussion of c-command)? How does the parser “know where it is” to carry out these computations?

### Summary

I have argued that any model of language computation must answer both *how* and *why* questions, and that the ideal model should be a fusion of mechanistic and probabilistic elements. I have sketched a framework for language processing based on the psychophysiological mechanism of cue integration. The cue integration framework asserts a mechanistic psychological operation over probabilistic representations, which are represented by neural population codes that are flexibly combined using two simple canonical neural computations: summation and normalization. Together these operations comprise the cue integration mechanism. By restricting computation to canonical neural mechanisms, cue integration may be able to form a linking hypothesis between psycholinguistic, computational, and neurobiological theories of language.

The heart of this mechanistic claim is that the relationship between a given level and the next level of representation (between cue and “target”) is probabilistic, and that, in turn, this uncertainty forms a vital aspect of representation, incorporated via the cue integration mechanism of normalization. The main representational hypothesis of cue integration is that every level of representation is a cue to higher levels of representation, resulting in a cascaded architecture where activation can spread before processing of the current set of features completes. Crucially, cue integration can take as its input an endogenously stimulated representation and output another representational state, allowing all levels of linguistic input to be extracted from sensory input. While cues determine which representation is activated, cue reliabilities determine the strength of the evidence for a particular representation and thus how good of a model of the world the system has. Reliabilities reflect local processing context as well as global knowledge. Cue integration accounts for processing variables either by modulating the information expressed in the cue reliabilities, or by modulating the circuit of representations, in other words, changing the order of cue computations.

To close, the main criticism laid out in the first part of this article can of course be applied to the cue integration hypothesis: a central challenge for the cue integration model is to achieve a parsimony of cues, reliabilities, and population codes while preserving explanatory satisfaction.

## Author contributions

The author confirms being the sole author and contributor to this work and has approved it for publication.

### Conflict of interest statement

The author declares that the research was conducted in the absence of any commercial or financial relationships that could be construed as a potential conflict of interest.

## Footnotes

^1^In contrast to location-addressable systems, where data is stored irrespective of its content and a search must be executed to retrieve a particular target item.

^2^Cue-based retrieval interference, although some psycholinguistic work invokes the notion of *encoding* interference, whereby representations fail to be stably encoded when there are multiple similar items in memory (Hofmeister and Vasishth, 2014). However, the spirit of that notion is usually cached out as *proactiveinterference* in the recognition memory literature, whereby forgetting occurs due to information learned or encoded prior to the onset of the study item, but is still due to cue-overload (Anderson and Neely, 1996; Öztekin and McElree, 2007; Martin and McElree, 2009; Van Dyke and McElree, 2011; Van Dyke and Johns, 2012). Retroactive interference refers to forgetting due to information acquired after the onset of the study item (Gillund and Shiffrin, 1984; Anderson and Neely, 1996; McElree, 2006).

^3^Summation of the activation of the neural population tuned to a given stimulus or feature of the environment.

^4^Though the highest levels of hierarchical representation are reached via an arguably single modality in phone conversations, sign language, and reading—it is an empirical question as to whether processing in these cases activates linked representations generated from other modalities.

^5^Such an assertion then attributes most of the burden (and magic) of online language processing onto language acquisition. In a system of representation where only cues and their reliabilities are computed to activate the next representation, it is this existing knowledge that parses input and links up the right representations properly. But how in the world are these all important, pre-existing, parse-making representations acquired? See Section Cue Integration in Language Acquisition and Bilingualism for more discussion but no definitive answers.

^6^Also in feedback or top-down connections, which Singer (2013) claims are more numerous in neocortex than feed-forward connections.

^7^Out of fear, and for simplicity's sake, I am skipping how acoustic representations are transduced into phonetic and then phonemic representations.

^8^Along with the notion of search, both theories of grammar and processing often tacitly assume buffers and maintenance in the architectures they imply.

^9^By “percolate” I mean persist in being represented or coded in active neural populations as processing proceeds.

^10^Surround suppression is a characteristic of neurons in primary visual areas wherein a given neuron's activity is reduced in the presence of a stimulus outside its receptive field; lateral inhibition from neurons with different receptive fields is one possible mechanism through which surround suppression may arise (Xing and Heeger, 2000).

^11^Note the similarity in time-based mechanisms with Bornkessel-schlesewsky et al. (2015) and Giraud and Poeppel (2012), and similar to the notion of noise correlation in population coding put forth by Averbeck et al. (2006).
